# Oxidative-Stress-Mediated AMPK/mTOR Signaling in Bovine Mastitis: An Integrative Analysis Combining 16S rDNA Sequencing and Molecular Pathology

**DOI:** 10.3390/biology15020115

**Published:** 2026-01-06

**Authors:** Yuanyuan Zhang, Min Zhang, Daqing Wang, Feifei Zhao, Luofei Jia, Zhiwei Sun, Guifang Cao, Yong Zhang

**Affiliations:** 1 College of Veterinary Medicine, Inner Mongolia Agricultural University, Hohhot 010018, China; zyyworkaccount@163.com (Y.Z.); zhangmin5400@126.com (M.Z.); wangdaqing050789@126.com (D.W.); imauzff@126.com (F.Z.); 18713193970@163.com (L.J.); 2 Animal Embryo and Developmental Engineering Key Laboratory of Higher Education, Institutions of Inner Mongolia Autonomous Region, Hohhot 010018, China; 3 Inner Mongolia Autonomous Region Key Laboratory of Basic Veterinary Medicine, Hohhot 010018, China; 4 Tongliao Institute of Agricultural and Animal Husbandry Sciences, Tongliao 028000, China; sunzhiwei4777@163.com; 5 College of Life Sciences, Inner Mongolia University, Hohhot 010021, China; 6 National Dairy Industry Technology Innovation Center, Hohhot 010010, China

**Keywords:** dairy cow, mammary gland, bacterial identification, oxidative stress, immunohistochemistry

## Abstract

The bovine mammary gland, the exclusive site of milk synthesis, is a structurally specialized tissue that houses distinct cellular subsets, yet it remains highly susceptible to major mastitis pathogens, including *Staphylococcus aureus*, *Streptococcus agalactiae*, and *Escherichia coli*. Infection disrupts redox homeostasis, leading to excessive accumulation of reactive oxygen species (ROS) and rapid activation of antioxidant pathways. In this study, we integrated 16S DNA sequencing, histopathology (hematoxylin and eosin), and immunohistochemistry to map the mastitis-associated microbiota and visualize oxidative-damage foci in mammary tissues challenged by *Staphylococcus aureus*, *Streptococcus agalactiae*, or *Escherichia coli*. Quantitative reverse transcription polymerase chain reaction and Western blot analyses were subsequently performed on the same samples to measure the kinetic response of six oxidative-stress-related signalling nodes: adenosine 5′-monophosphate-activated protein kinase, cytochrome P450 1A1, heme oxygenase 1, nitric oxide synthase, mammalian target of rapamycin, and superoxide dismutase. By correlating the temporal expression patterns of these genes/proteins with ROS accumulation and histological severity, this study delineates the molecular cascade linking oxidative imbalance to mastitis pathology, providing data-driven targets for future preventive and therapeutic strategies.

## Simple Summary

Bovine mastitis is a costly udder disease initiated by bacterial infection and exacerbated by oxidative stress. In this study, we integrated bacterial sequencing, tissue staining, and molecular assays to map pathogens, lesion stages, and markers of oxidative-stress signaling (adenosine 5′-monophosphate-activated protein kinase, cytochrome P450 1A1, heme oxygenase 1, mammalian target of rapamycin, nitric oxide synthase, and superoxide dismutase) in 14 mastitic glands. *Escherichia coli*, *Aeromonas*, and *Pseudomonas* were predominant, driving a progressive transition from acute necrosis to chronic fibro-calcific damage. Distinct “oxidative-stress signatures” were identified as potential early biomarkers and therapeutic targets for antioxidant-based interventions. These findings provide a practical “bacteria–pathology–molecule” framework for precise mastitis control in dairy cows.

## 1. Introduction

Based on observable changes in the udder and milk, bovine mastitis is classified into clinical and subclinical (latent) mastitis. The incidence of clinical mastitis typically ranges from 2% to 5% [1]. Affected cows exhibit visible and palpable signs of udder inflammation, including swelling, pain, redness, and impaired function, ultimately leading to reduced milk yield. In severe cases, systemic signs may develop, such as fever, altered milk appearance and volume, inappetence, and recumbency. In the most extreme situations, milk production ceases, and the cow may become completely anorexic. Subclinical mastitis, also referred to as latent mastitis, is an inflammatory condition of the mammary tissue without overt clinical signs. Globally, its average prevalence is 30–50% [2]. Although asymptomatic, it exerts several detrimental effects on both milk and the affected cow. Essential amino acids and other beneficial milk components decline sharply, and the thermal stability of milk is reduced [3]. Concurrently, blood zinc concentrations decrease markedly in affected cows [4]. Because subclinical mastitis often remains undetected and underappreciated, its impact on dairy products is easily underestimated. Its prolonged course and frequent multidrug resistance contribute to a progressive decline in both milk volume and quality, ultimately reducing farm profitability. Bovine mastitis is caused by a variety of pathogens [5]. The principal causative agents include *Streptococcus uberis*, *Staphylococcus aureus*, *Escherichia coli*, *Streptococcus agalactiae*, *Streptococcus dysgalactiae*, and *Mycoplasma* spp. In the farm environment, *Staphylococci*, *Coliforms*, and *Streptococci* are ubiquitous and most behave as opportunistic pathogens [6,7,8]. When husbandry or milking management is inadequate, these organisms readily invade the udder and may spread quickly through milking equipment and other fomites [9]. Modern dairy production is increasingly shifting from blanket dry-cow antibiotic therapy to selective dry-cow therapy, in which only cows with high somatic cell counts or a history of mastitis receive antimicrobials [10].

Oxidative stress in living organisms arises when the balance between oxidant and antioxidant systems is disrupted by harmful stimuli, shifting the equilibrium toward oxidation and generating excessive reactive oxygen species (ROS) that damage cells and tissues [11]. Under oxidative conditions, lipids and other macromolecules are readily oxidized, producing lesions that alter the structure and function of cell membranes and other cellular components [12]. Studies have demonstrated that oxidative stress can modify physiological functions and precipitate pathological injury. ROS play critical biological roles: they are unavoidable by-products of cellular respiration and protein folding, as well as end products of numerous metabolic reactions [13]. The primary intracellular sources of ROS are mitochondria and NADPH oxidases (NOX) [14]. Although ROS are normal metabolites of viable cells, oxidative stress activates multiple transcription factors, leading to differential gene expression in inflammatory pathways. Oxidative stress-driven inflammation underlies many chronic diseases. Excessive ROS directly damages lipids, proteins, and DNA, thereby triggering inflammatory responses that further intensify oxidative stress [15]. During inflammation, activated immune cells release additional ROS, further increasing oxidative load. Thus, oxidative stress and inflammation act synergistically to initiate and propagate cardiovascular and respiratory disorders [16].

Oxidative stress is recognized as a metabolic disorder that affects entire organ systems. Its presence not only compromises animal health but also diminishes the quality of final products; for instance, vitamins A, E, and C, and polyunsaturated fatty acids are degraded during oxidative stress, thereby affecting milk quality [17]. In dairy cows, oxidative stress reduces milk yield at all stages of lactation [18]. Under physiological conditions, normal mitochondrial metabolism generates potentially damaging levels of ROS, which are usually neutralized by enzymatic and non-enzymatic antioxidant defences. However, when disease, inflammation, or cytotoxic factors intervene, ROS continuously escape from mitochondria into the cytosol, damaging DNA, proteins, and lipids [19]. Cows possess an endogenous antioxidant network capable of counteracting and repairing oxidative injury within limits [20]. Once this threshold is exceeded, a full oxidative stress response is initiated. Oxidative stress is now recognized as a key driver of mastitis pathogenesis in dairy cows. Intramammary infection triggers an explosive respiratory burst in resident neutrophils and macrophages, generating surplus ROS that overwhelm local antioxidant defenses. This redox imbalance not only damages lipid bilayers, mitochondrial DNA and tight-junction proteins, thereby compromising blood–milk barrier integrity, but also amplifies the inflammatory cascade by activating NF-κB and MAPK axes. Studies show that supplementing transition cows with N-carbamylglutamate enhances neutrophil function while reducing inflammation and oxidative stress [21]. In healthy cells, the expression levels of adenosine 5′-monophosphate-activated protein kinase (AMPK), cytochrome P450 1A1 (CYP1A1), and HMOX-1 are low and maintained at basal levels to preserve redox homeostasis. Under stress conditions, such as oxidative stress or inflammation, HMOX-1 expression increases markedly. Nitric oxide synthase (NOS) is also expressed at low levels, primarily in endothelial, neuronal, and macrophage cells. Mammalian target of rapamycin (mTOR) activity is normally maintained at basal levels, whereas SOD expression is low under physiological conditions but increases in response to oxidative stress. Upregulating antioxidant/anti-inflammatory pathways, such as superoxide dismutase (SOD) and heme oxygenase 1 (HMOX-1), helps preserve udder health while meeting antimicrobial-stewardship requirements, providing a practical context for studying homeostasis in bovine mammary epithelial cells under reduced-antibiotic conditions. The energy-sensing AMPK pathway and its negative regulator mTOR are simultaneously perturbed during this process: AMPK activation suppresses NLRP3 inflammasome assembly and promotes Nrf2-driven antioxidant gene expression, whereas sustained mTOR activity sustains pro-inflammatory cytokine translation and neutrophil infiltration. Thus, oxidative insults and the reciprocal interplay between AMPK and mTOR appear to form a feed-forward loop that governs the intensity and duration of mastitis.

This study aims to identify the bacterial pathogens infecting the mammary tissue of dairy cows and to characterize the histopathological alterations induced by oxidative stress. By doing so, it lays the groundwork for elucidating the complex pathogenesis of bovine mastitis and identifying novel targets for its prevention and control.

## 2. Materials and Methods

### 2.1. Main Materials

Bovine mammary gland tissue and udder exudate were collected from a slaughterhouse near Hohhot, Inner Mongolia. During slaughter, cattle underwent a brief clinical inspection. Only those exhibiting reddened and swollen udders, increased firmness, and abnormal milk appearance (yellowish, viscous, or clotted) were selected for sampling. After identification, tissue and exudate were collected from the affected quarters. Two approximately 1 cm^3^ fragments of mammary tissue were aseptically excised using sterile instruments: one piece was snap-frozen in liquid nitrogen, and the other was fixed in paraformaldehyde. Udder exudate was aspirated and aliquoted into sterile centrifuge tubes. A total of 13 mammary gland tissue and exudate samples were collected. All animal experiments were conducted in accordance with the Administration of Affairs Concerning Experimental Animals in China. The experimental protocol was approved by the Animal Welfare and Research Ethics Committee of Inner Mongolia Agricultural University (Approval ID: NND202103).

### 2.2. Main Reagents

Environmentally friendly dewaxing transparent liquid, neutral paraformaldehyde fixative, hematoxylin–eosin (H&E) HD constant dye kit, 20× citric acid antigen retrieval solution (pH 6.0), 20× Tris-EDTA antigen retrieval solution (pH 9.0), 20× Tris-EDTA antigen retrieval solution (pH 8.0), universal neutral tissue fixative, phosphate-buffered saline (PBS), bovine serum albumin (BSA), normal rabbit serum (concentrated), hematoxylin staining solution, hematoxylin differentiation solution, hematoxylin bluing reagent solution, super-clean quick-drying sealing adhesive, and DAB chromogenic reagent for immunohistochemistry were purchased from Servicebio (Wuhan, China). The Ezup columnar bacterial genomic DNA extraction kit and SanPrep columnar DNA gel recovery kit were purchased from Sangon Biotech (Shanghai, China). Other common reagents were purchased from Sinopharm Chemical Reagent Co., Ltd. (Shanghai, China).

### 2.3. Main Instruments

The embedding machine and freezing platform were purchased from Wuhan Junjie Electronics Co., Ltd. (Wuhan, China). The dehydrator was purchased from DIAPATH (Martinengo, Italy). The tissue spreader was purchased from Zhejiang Kehua Instrument Co., Ltd. (Hangzhou, China)., and the pathology slicer from Shanghai Leica Instrument Co., Ltd. (Shanghai, China). The oven was purchased from Tianjin Laibo Rui Instrument Equipment Co., Ltd. (Tianjin, China). Adhesive slides for paraffin sections were purchased from Servicebio (Wuhan, China), and cover glasses from Citotest Labware Manufacturing Co., Ltd. (Nanjing, China). The biosafety cabinet was purchased from Jiangsu Sujie Chemical Equipment Factory (Changshu, China). The upright optical microscope and imaging system were purchased from Nikon (Tokyo, Japan). The polymerase chain reaction (PCR) amplification instrument was purchased from BBI (Shanghai, China). The high-speed microcentrifuge was purchased from Sangon Biotech (Shanghai, China) Co., Ltd. The electrophoresis system was purchased from Beijing Liuyi Biotechnology Co., Ltd. (Beijing, China). The microspectrophotometer was purchased from Merinton Instrument, Inc. (Ann Arbor, MI, USA). The Roche GS FLX sequencer was purchased from ABI (Carlsbad, CA, USA).

### 2.4. Experimental Methods

#### 2.4.1. Collection and Fixation of Materials

Two approximately 1 cm^3^ fragments of mammary tissue were aseptically excised: one was snap-frozen in liquid nitrogen for RNA and protein extraction, and the other was fixed in paraformaldehyde for histological sectioning. Exudate samples were plated onto various culture media for subsequent bacterial isolation and identification.

#### 2.4.2. Bacterial Isolation and Identification

The collected exudates were separately plated onto MacConkey agar, Luria-Bertani (LB) agar, blood agar, and nutrient broth, and then incubated at 37 °C for 15 h. Well-isolated single colonies were transferred into LB medium for preservation and subsequently sent to Sangon Biotech (Shanghai) Co., Ltd. for 16S ribosomal DNA (16S rDNA) identification. The 16S rDNA identification procedure included the following steps: extraction of total bacterial DNA and assessment of DNA quality; amplification of the 1.5 kb full-length 16S rRNA gene using universal primers 27F/1492R; verification of PCR products by agarose gel electrophoresis followed by purification; Sanger bidirectional sequencing; assembly of high-quality reads using Chromas; and sequence comparison using NCBI-BLASTn (BLAST+ 2.17.0). Sequence identity ≥99% was assigned to the same species, and 97%–99% to the same genus. The final output included species name, sequence similarity, accession number, and a phylogenetic tree, with the original chromatograms and FASTA files retained for reference. The primers used were as follows:

27F: AGAGTTTGATCMTGGCTCAG

1492R: GGTTACCTTGTTACGACTT

#### 2.4.3. H&E Staining Procedures

Paraffin sections were dewaxed and rehydrated through eco-friendly dewaxing solutions I and II (20 min each), absolute ethanol I and II (5 min each), 75% ethanol (5 min), and rinsed with tap water. Frozen sections were equilibrated from −20 °C to room temperature, fixed for 15 min, and rinsed. All sections were pretreated with HD Constant Stain for 1 min, stained with hematoxylin for 3–5 min, rinsed with tap water, differentiated, blued, and finally rinsed. Sections were dehydrated in 95% ethanol for 1 min, counterstained with eosin for 15 s, and progressively dehydrated through absolute ethanol I–III and n-butanol I–II for 2 min each. After clearing in xylene I–II for 2 min each, sections were mounted with neutral balsam and examined microscopically for imaging and analysis.

#### 2.4.4. Immunohistochemical Experiment Procedures

Paraffin sections were dewaxed and rehydrated using eco-friendly dewaxing solutions I–III (10 min each), followed by absolute ethanol I–III (5 min each). After rinsing in distilled water, antigen retrieval was performed under tissue-appropriate conditions, avoiding evaporation. Sections were allowed to cool and then washed three times in PBS (5 min each). Endogenous peroxidase activity was blocked with 3% hydrogen peroxide (H_2_O_2_) for 25 min, followed by PBS washes. Non-specific binding sites were blocked using 3% BSA (or rabbit serum for goat primary antibodies) for 30 min. The primary antibody diluted in PBS was applied and incubated overnight at 4 °C. After washing, slides were incubated with a horseradish peroxidase (HRP)-conjugated, species-matched secondary antibody for 50 min at room temperature. Slides were washed again, developed with freshly prepared DAB until a brown-yellow reaction appeared, then rinsed. Nuclei were counterstained with hematoxylin (3 min), differentiated, blued, and rinsed. Sections were dehydrated through graded alcohols (75%, 85%, absolute ethanol I–II, 5 min each), cleared in n-butanol (5 min) and xylene I (5 min), air-dried, and mounted. Final evaluation was performed under a bright-field microscope.

Immunohistochemical results were analysed using Aipathwell^®^ (2.2.4) software (Servicebio^®^). First, the tissue was localized, and the region of interest was delineated. Using the Hue, Saturation, and Intensity colour model, the software automatically detected positive staining and classified it into three levels: weak positive (light yellow; score 1), moderate positive (brownish yellow; score 2), and strong positive (dark brown; score 3). Depending on the analysis requirements, the software identified cell nuclei, expanded the cytoplasmic region, and quantified the number and area of weakly, moderately, and strongly positive cells. It also calculated several parameters, including integrated optical density (IOD) and total tissue area. The region of interest was examined systematically under high magnification, after which the software automatically computed all metrics and generated the final results based on the raw data and algorithmic formulas. Immunohistochemistry results were evaluated using parameters related to the number of positive cells. The positive cell ratio, calculated as the number of positive cells divided by the total number of cells, reflects the proportion of positive cells within a relatively homogeneous cell population. The H-score (Histochemistry score) is a semi-quantitative system that incorporates both the percentage of positive cells and staining intensity into a single value: H-score = Σ(pi × i) = (% weak-positive cells × 1) + (% moderate-positive cells × 2) + (% strong-positive cells × 3). Intensity levels (i) are defined as: 0 = negative (no staining), 1 = weak (light yellow), 2 = moderate (brown-yellow), and 3 = strong (dark brown). Pi represents the percentage of cells at each intensity level. H-scores range from 0 to 300, with higher valuesindicating stronger and more extensive staining. The Immunoreactive Score (IRS) is calculated as SI (staining intensity) × PP (percentage of positive cells). SI is graded as: 0 = no staining, 1 = weak (light yellow), 2 = moderate (brown-yellow), 3 = strong (dark brown). PP is graded as: 0 = 0%–5%, 1 = 6%–25%, 2 = 26%–50%, 3 = 51%–75%, 4 = >75% positive cells. Higher IRS values indicate stronger combined staining intensity and positivity. Positive cell density, defined as the number of positive cells per unit tissue area, reflects the distribution and abundance of a given cell type within the tissue. Mean optical density (IOD/area of positive pixels) represents the average staining intensity and is commonly used to assess signal strength.

#### 2.4.5. Total RNA Extraction and Quantitative Reverse Transcription Ploymerase Chain Reaction (RT-qPCR) Analysis

Total RNA was extracted from bovine mammary tissue using an AXYGEN kit (AXYGEN, Tuxbury, MA, USA), following the manufacturer’s instructions. Reverse transcription was performed using the PrimeScript RT Reagent Kit (Vazyme, Wuhan, China) at 37 °C for 15 min, followed by 85 °C for 5 s. RNA concentration and purity (A260/A280) were measured using a microplate reader (BioTek, Winooski, VT, USA); acceptable ranges were 500–1000 ng/μL and 1.8–2.0, respectively. Complementary DNA was stored at −80 °C. β-actin was used as the endogenous control gene. Quantitative PCR conditions were: 50 °C for 2 min; 95 °C for 10 min; followed by 95 °C for 15 s; and 60 °C for 60 s. Reactions were prepared according to the TB^®^ Green protocol. Primers for β-actin, AMPK, CYP1A1, HMOX-1, NOS, mTOR, and SOD were designed and synthesized by Shengong Biological Co., Ltd. (Shanghai, China). Primer sequences are shown in Table 1.

#### 2.4.6. Western Blot

Total cellular proteins were extracted according to the instructions of the total protein extraction kit. Proteins were separated on 10% sodium dodecyl sulphate-polyacrylamide gel electrophoresis (SDS-PAGE) and transferred to nitrocellulose membranes. Membranes were blocked with 5% BSA at room temperature for 4 h and incubated overnight at 4 °C with primary antibodies against β-actin, AMPK, CYP1A1, HMOX-1, NOS, mTOR, and SOD. After washing five times with tris—buffered saline with Tween-20, membranes were incubated with HRP-conjugated goat anti-rabbit or goat anti-mouse immunoglobulin G for 1 h at room temperature. Protein bands were visualized using enhanced chemiluminescence and analysed via ImageJ (1.48V) grayscale quantification.

### 2.5. Data Processing and Analysis

**Scheme 1:**
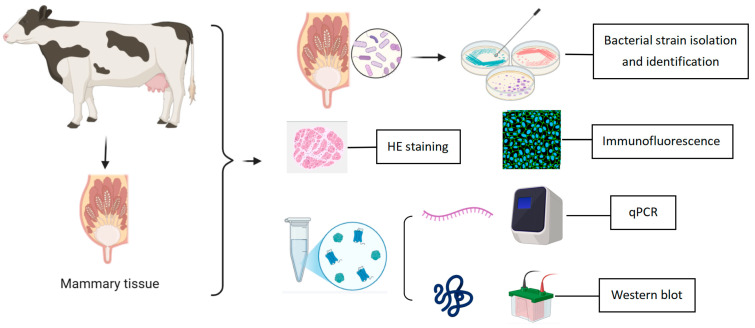
Experimental Method Flowchart.

Data were analysed using the 2^−ΔCt^ method. GraphPad Prism 8 was used for plotting and two-way analysis of variance. A significance level of * *p* < 0.05 was considered statistically significant, while ** *p* < 0.01 and *** *p* < 0.001 were considered highly significant. In our study, each experiment was conducted with three biological replicates to ensure the reliability and reproducibility of the results. The data presented in the article are expressed as mean ± standard deviation to reflect data variability. The flowchart of the research content is as follows (Scheme 1).

## 3. Results

### 3.1. Bacterial Isolation, Cultivation, and Strain Identification

**Figure 1:**
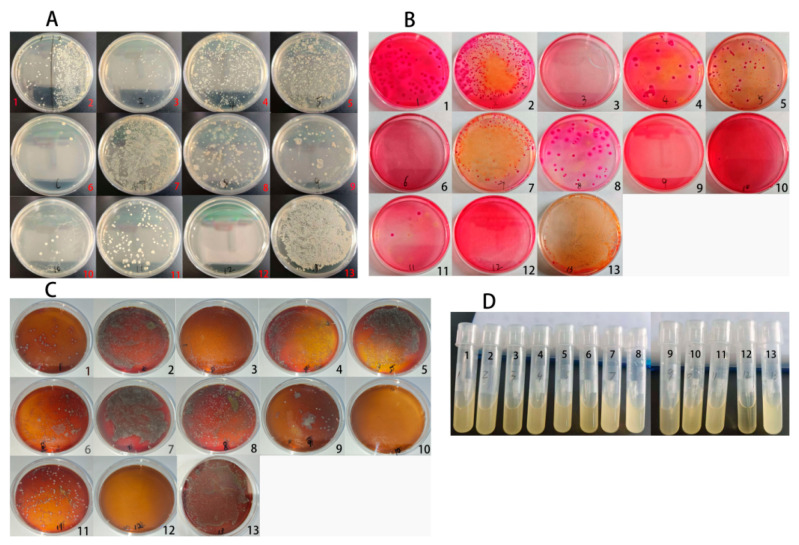
Bacterial isolation and culture. (**A**) Colonies were isolated and cultured on Luria-Bertani agar. (**B**) Colonies isolated and cultivated on MacConkey agar. (**C**) Colonies are isolated and cultured on blood agar. (**D**) Bacteria cultivated in nutrient broth. The numbers 1–13 indicate that bacteria isolated and cultured from 13 different samples.

The results of bacterial isolation from exudates using different culture media are shown in Figure 1, Table 2. Samples for bacterial isolation and identification were collected from the milk and exudates of 13 cows. Bacteria were successfully isolated from all cows, and more than one bacterial species was isolated from eight of them. *Escherichia coli* was isolated from samples 1 and 2; *Pseudomonas* and *Acinetobacter johnsonii* from sample 3; *Escherichia coli*, *Pseudomonas*, and *Aeromonas* from sample 4; *Escherichia coli* and *Aeromonas* from sample 5; *Pseudomonas peli* and *Comamonas* from sample 6; *Escherichia coli* and *Aeromonas salmonicida* from sample 7; *Escherichia coli* and *Pseudomonas* from sample 8; *Bacillus licheniformis* from sample 9; *Aeromonas* from sample 10; *Pseudomonas guangdongensis*, *Staphylococcus haemolyticus*, and *Mammaliicoccus sciuri* from sample 11; no single colonies from sample 12; and *Escherichia coli* and *Klebsiella pneumoniae* from sample 13.

### 3.2. H&E Staining

**Figure 2:**
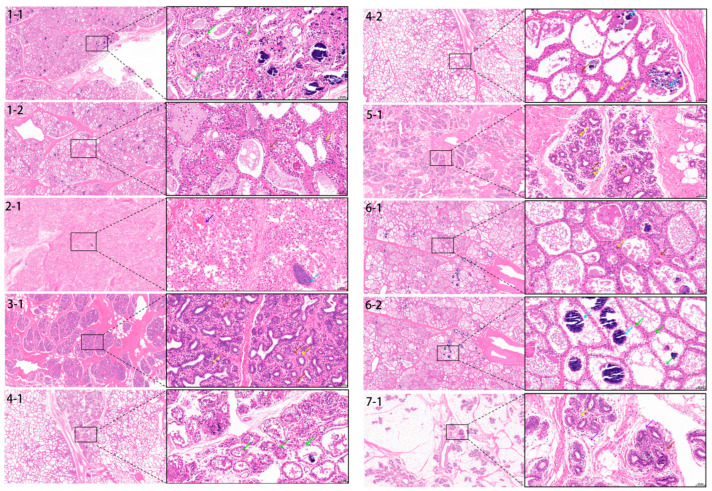

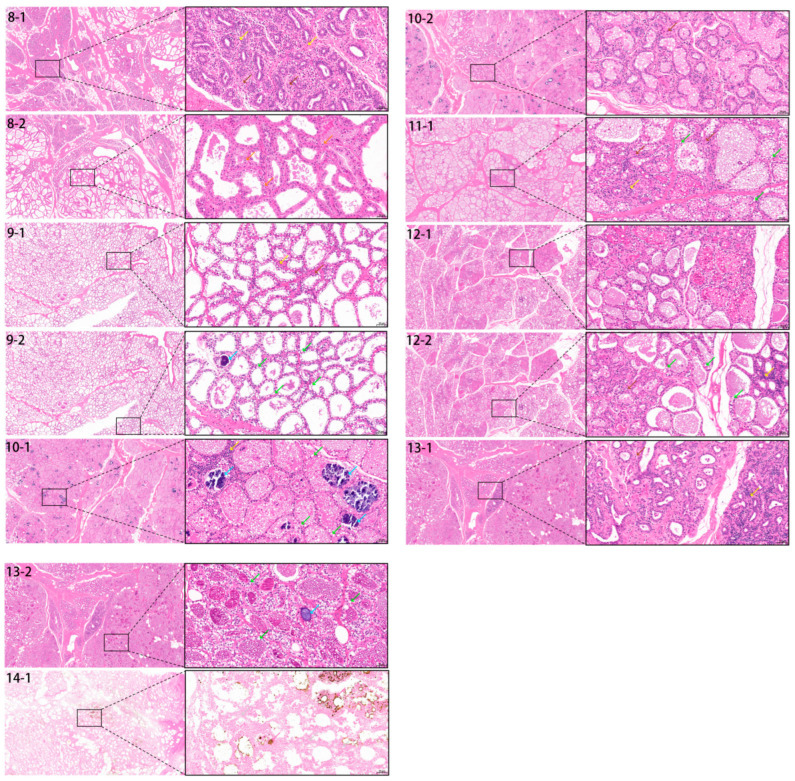
Results of hematoxylin–eosin staining of 14 breast tissue samples. Silver arrow: cellular necrosis. Cyan arrow: calcification focus. Blue arrow: hemorrhage. Green arrow: intracellular cytoplasmic vacuoles. Brown arrow: connective tissue hyperplasia. Yellow arrow: lymphocytic infiltration. Black box: location of the magnified field of view. Numbers 1–14 indicate that the 14 tissues subjected to HE staining. Scale bar = 50 μm.

The histopathological section shown in Figure 2(1-1) reveals abundant alveoli in the mammary tissue. Numerous alveolar epithelial cells exhibit round cytoplasmic vacuoles of variable size (green arrows). Figure 2(1-2) shows that a few alveolar epithelial cells are necrotic and sloughed, with pyknotic, deeply stained nuclei (silver arrows). Mild fibroplasia is present in the stroma (brown arrows), accompanied by focal lymphocytic infiltrates (yellow arrows). In Figure 2(2-1), extensive necrosis of alveolar epithelial cells is observed; the nuclei are pyknotic, fragmented, or dissolved (silver arrows), and the normal architecture is disrupted. Occasional intra-alveolar calcifications (cyan arrows) and rare haemorrhage (blue arrows) are also noted. Figure 2(3-1) shows numerous but small alveoli, and the stroma displays marked fibroplasia (brown arrows) with scattered lymphocytes and focal granulocytic infiltrates (yellow arrows).

Figure 2(4-1) shows abundant alveoli, and many alveolar epithelial cells contain round cytoplasmic vacuoles of various sizes (green arrows). Figure 2(4-2) shows that a few alveolar epithelial cells are necrotic and sloughed, with pyknotic, deeply stained nuclei (silver arrows). Multiple intra-alveolar calcifications (cyan arrows), mild stromal fibroplasia (brown arrows), and focal lymphocytic infiltrates (yellow arrows) are present. In Figure 2(5-1), the mammary tissue contains abundant but small alveoli. Prominent stromal fibroplasia (brown arrows) is accompanied by diffuse lymphocytic and granulocytic infiltrates (yellow arrows), mild edema, and loosely arranged connective tissue (purple arrows). Figure 2(6-1) shows numerous alveoli with multifocal stromal fibroplasia (brown arrows) and focal lymphocytic infiltrates (yellow arrows). In Figure 2(6-2), many alveolar epithelial cells exhibit round cytoplasmic vacuoles of variable size (green arrows), and abundant intra-alveolar calcifications are observed (cyan arrows). Figure 2(7-1) shows abundant small alveoli; a few epithelial cells are necrotic with pyknotic, deeply stained nuclei (silver arrows). Multifocal stromal fibroplasia (brown arrows), scattered lymphocytic infiltrates (yellow arrows), extensive edema with loosely arranged connective tissue (purple arrows), and large areas of adipocytes are also present.

Figure 2(8-1), the mammary tissue contains abundant alveoli; noticeable stromal fibroplasia (brown arrows) is accompanied by scattered lymphocytic and granulocytic infiltrates (yellow arrows). Figure 2(8-2) shows numerous alveoli, and many alveolar epithelial cells exhibit hydropic degeneration—cells are swollen with loose, lightly stained cytoplasm (orange arrows). Figure 2(9-1) demonstrates abundant alveoli with multifocal mild stromal fibroplasia (brown arrows) and focal lymphocytic infiltrates (yellow arrows. Figure 2(9-2) shows that many alveolar epithelial cells contain round cytoplasmic vacuoles of variable size (green arrows), and occasional intra-alveolar calcifications are present (cyan arrows).

Figure 2(10-1) shows plentiful alveoli; numerous alveolar epithelial cells display round cytoplasmic vacuoles of varying sizes (green arrows), abundant intra-alveolar calcifications (cyan arrows), and focal, sparse lymphocytic infiltrates (yellow arrows). Figure 2(10-2) shows focal stromal fibroplasia (brown arrows). Figure 2(11-1) shows mammary tissue with abundant alveoli. Numerous alveolar epithelial cells contain round cytoplasmic vacuoles of varying sizes (green arrows), and multifocal stromal fibroplasia (brown arrows) is accompanied by sparse lymphocytic infiltrates (yellow arrows). Figure 2(12-1) shows abundant alveoli with necrotic cellular debris present in multiple alveoli (silver arrows). Figure 2(12-2) shows many alveolar epithelial cells displaying round cytoplasmic vacuoles of variable size (green arrows), together with multifocal stromal fibroplasia (brown arrows) and lymphocytic infiltrates (yellow arrows).

Figure 2(13-1) shows mammary tissue with plentiful alveoli. Focal stromal fibroplasia (brown arrows) is associated with prominent lymphocytic infiltrates (yellow arrows). Figure 2(13-2) shows numerous alveolar epithelial cells containing round cytoplasmic vacuoles of various sizes (green arrows) and multiple intra-alveolar calcifications (cyan arrows). Figure 2(14-1) shows extensive necrosis and lysis of parenchymal cells (silver arrows) with indistinct tissue architecture. Abundant brownish-yellow material is deposited (pink arrows), and no significant inflammatory infiltrate is observed.

### 3.3. Immunohistochemistry

**Figure 3:**
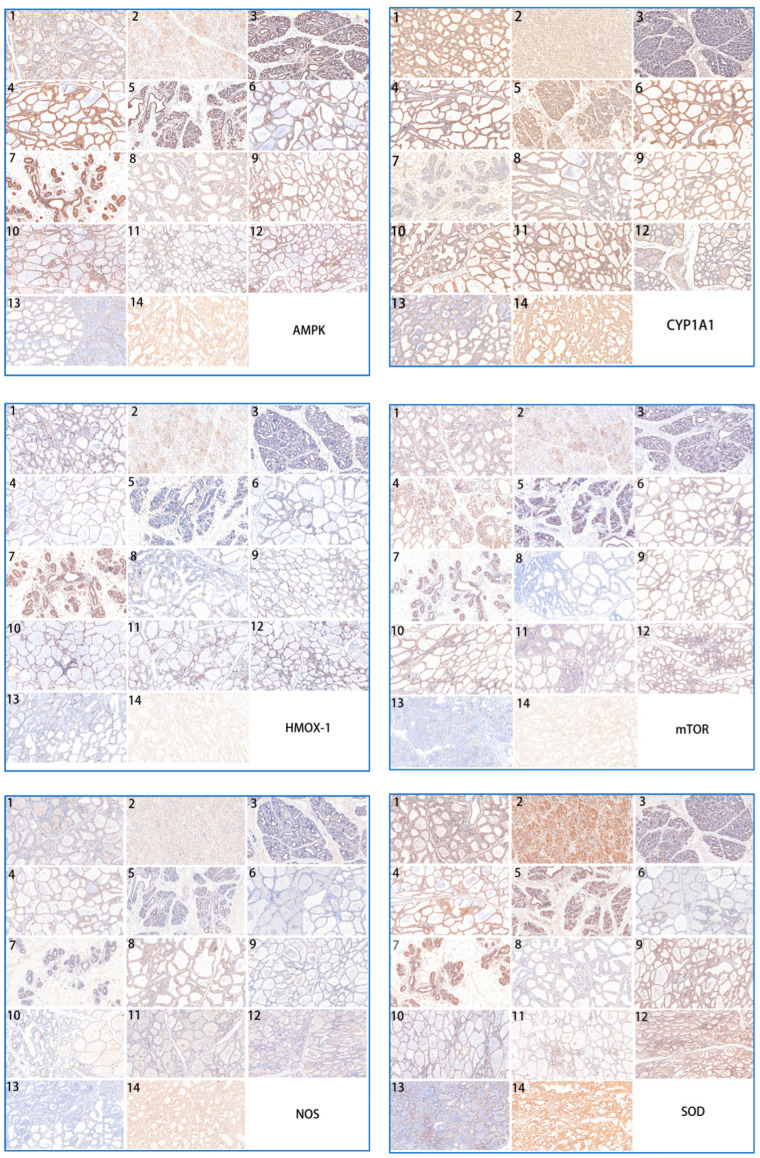
Immunohistochemical results for adenosine 5′-monophosphate-activated protein kinase, cytochrome P450 1A1, heme oxygenase 1, mammalian target of rapamycin, nitric oxide synthase, and superoxide dismutase in 14 tissues. Numbers 1–14 denote the cow mammary-gland tissue samples.

Only qualitative detection was performed; no comparative analysis among the six proteins was conducted. Because tissue 14 showed severe necrosis and a very low cell count, quantitative analysis of positive cells was not possible(Table 3, Figure 3).

### 3.4. Expression Levels of Oxidative Stress-Related Genes in Bovine Mammary Tissue

**Figure 4:**
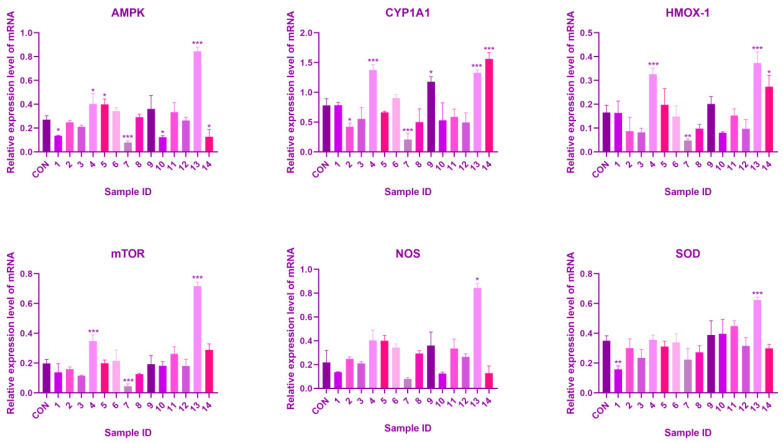
Quantitative polymerase chain reaction results for the 14 tissues showing messenger RNA expression levels of adenosine 5′-monophosphate-activated protein kinase, cytochrome P450 1A1, heme oxygenase 1, mammalian target of rapamycin, nitric oxide synthase, and superoxide dismutase (* *p* < 0.05; ** *p* < 0.01; and *** *p* < 0.001).

Mammary tissue without mastitis was used as the control group. Compared with the control, *AMPK* messenger RNA (mRNA) transcription levels showed a significant decrease in tissues 1, 10, and 14 (*p* < 0.05); a significant increase in tissues 4 and 5 (*p* < 0.05); a highly significant decrease in tissue 7 (*p* < 0.001); and a highly significant increase in tissue 13 (*p* < 0.001).*CYP1A1* mRNA levels showed a significant decrease in tissue 2 (*p* < 0.05); a significant increase in tissue 9 (*p* < 0.05); a highly significant decrease in tissue 7; and highly significant increases in tissues 4, 7, 13, and 14 (*p* < 0.001). *HMOX-1* mRNA transcription levels were significantly increased in tissue 14 (*p* < 0.05), markedly decreased in tissue 7 (*p* < 0.01), and highly significantly increased in tissues 4 and 13 (*p* < 0.001). *mTOR* mRNA transcription levels showed a highly significant decrease in tissue 7 and a highly significant increase in tissues 4 and 13 (*p* < 0.001). *NOS* mRNA transcription levels increased significantly in tissue 13 (*p* < 0.05). *SOD* mRNA transcription levels were markedly decreased in tissue 1 (*p* < 0.01) and highly significantly increased in tissue 13 (*p* < 0.001) (Figure 4).

### 3.5. Western Blot

**Figure 5:**
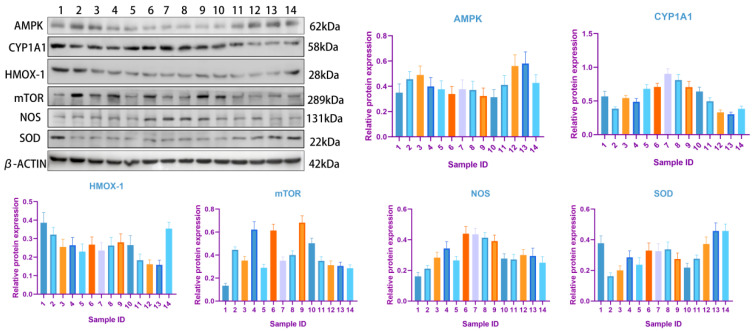
Western blot results for the 14 tissues showing protein expression levels of adenosine 5′-monophosphate-activated protein kinase, cytochrome P450 1A1, heme oxygenase 1, mammalian target of rapamycin, nitric oxide synthase, and superoxide dismutase. The original Western blot images can be found in the Supplementary Materials section (File S1) at the end of the manuscript.

Due to the 15-well limit of the SDS–PAGE gel, only 14 tissue samples could be loaded in addition to the protein marker; therefore, no control group was included. Protein expression levels across tissues were as follows: AMPK expression varied only slightly, with tissue 5 below and tissue 13 above the average. CYP1A1 expression was generally uniform, except for higher levels in tissue 1. HMOX-1 expression showed minimal variation, although tissues 1 and 13 were slightly above average. mTOR expression varied markedly: tissues 5, 7, 8, and 14 were below average, whereas tissues 2, 4, 6, 9, 10, 12, and 13 were slightly above. NOS protein expression also varied widely, with tissues 13 and 14 below average and tissues 1, 9, 11, and 12 slightly above average. SOD protein expression varied considerably, with tissues 1, 11, 12, and 13 above average(Figure 5).

## 4. Discussion

Although the technical levels of breeding, reproduction, and husbandry management have been continuously improving both domestically and internationally, the overall incidence rate of clinical mastitis remains as high as 33.4% [10]. Mastitis causes global economic losses exceeding 35 billion USD, driven by reduced milk production in lactating cows, increased breeding costs, decreased milk quality, reduced dairy-cow longevity, higher labour demands, and increased risks of disease transmission [22]. Minor mastitis-causing pathogens include *Corynebacterium* spp. and coagulase-negative *staphylococci*. Other pathogens include *Aerococcus viridans*, *Aerococcus* spp., *Enterococcus* spp., *Streptococcus salivarius*, *Streptococcus* spp., *Lactococcus* spp., *Pasteurella multocida*, *Mycoplasma bovis*, *Mycoplasma* spp., and *Prototheca* spp. [23].

16S rDNA identification refers to the sequencing of the bacterial 16S rDNA region for species-level identification [24]. The process involves bacterial genomic DNA extraction, PCR amplification using 16S rDNA–specific primers, purification of PCR products, DNA sequencing, and sequence alignment [25]. This approach provides rapid insight into bacterial species composition. The 16S rRNA is universally present in prokaryotes. rRNA plays an essential role in protein synthesis and exhibits a highly conserved function across all organisms. Moreover, it remains unchanged over the long course of biological evolution and can be regarded as a biological evolutionary clock. Because it contains both highly conserved and moderately conserved or hypervariable regions, the 16S rRNA molecule is suitable for examining phylogenetic relationships across organisms with varying evolutionary distances [26].

Histopathology (H&E staining) revealed a stepwise progression of mammary lesions: Stage 1—Metabolic disturbance (Figure 2(1-1,4-1,6-2,9-2,10-1,11-1,12-2,13-2): epithelial cytoplasm showing variably sized vacuoles indicating secretory arrest. Stage 2—Focal necrosis (Figure 2(1-2,4-2,7-1,12-1)): pyknotic and sloughed nuclei, mild fibroplasia, and lymphocytic infiltrates. Stage 3—Extensive necrosis and calcification (Figure 2(2-1,10-1,6-2,13-2)): nuclear fragmentation/lysis, abundant intra-alveolar dystrophic calcification, and petechial haemorrhage. Stage 4—Fibrotic repair (Figure 2(3-1,5-1,8-1,13-1)): alveolar atrophy, stromal collagen deposition, chronic inflammation, and edema. Stage 5—Hydropic change and adipose replacement (Figure 2(8-2,7-1)): swollen pale epithelium and adipocyte infiltration. Stage 6—Destruction (Figure 2(14-1)): complete parenchymal necrosis/lysis, brown granular deposits, and lack of inflammatory response. The histological lesions closely matched the bacteriological findings. *Escherichia coli* (samples 1, 2, 4, 5, 7, 8, and 13) drove the acute phase with extensive haemorrhagic necrosis and karyorrhexis (Figure 2(2-1,10-1)). *Aeromonas* spp. (samples 4, 5, 7, and 10) contributed to tissue liquefaction and subsequent dystrophic calcification. *Pseudomonas* spp. (samples 3, 4, 6, 8, and 11) sustained chronic inflammation through biofilm formation, corresponding to the notable fibroplasia and chronic infiltrates (Figure 2(3-1,5-1,8-1)). Low-virulence *Staphylococcus haemolyticus* (sample 11) elicited only sparse lymphoid foci (Figure 2(11-1,13-1)). *Bacillus licheniformis* (sample 9) represented secondary colonization, accompanied by mild fibrosis (Figure 2(9-1,9-2)). Polymicrobial infections (samples 4, 5, 7, 8, and 11) produced overlapping necrotic, calcific, and fibrotic patterns, confirming sequential lesion progression driven by bacterial synergy.

Overlaying mRNA and protein expression patterns revealed three dominant expression modes and two functional trajectories: Mode 1 (e.g., AMPK in tissue 13) shows a sharp rise in mRNA levels, accompanied by above-average protein levels, indicating synchronized upregulation and enhanced function. Mode 2 (e.g., CYP1A1-7) shows substantial mRNA decline while protein remains near baseline, indicating post-transcriptional compensation. Mode 3 (AMPK-1/10, CYP1A1-2, and HMOX1-7) exhibits marked mRNA reduction buffered by long-lived proteins, producing an “mRNA-down/protein-stable” pseudo-steady state. These modes form two biological narratives: Tissue 13 functions as an “anti-oxidative hotspot,” with coordinated activation of the AMPK–Nrf2–HMOX1/SOD pathway and moderate mTOR upregulation, fine-tuning autophagy–proliferation balance and maintaining high antioxidant capacity. Tissue 7 represents a “vulnerable zone,” where key transcripts are globally silenced, and proteins merely delay functional collapse; once protein reserves are depleted, rapid damage propagation is likely.

AMPK, a central regulator of cellular energy metabolism, provides crucial anti-oxidative protection in oxidative-stress-related diseases such as myocardial injury, neurodegeneration, and oocyte aging. It exerts these effects by activating transcription factors such as FOXO to increase antioxidant enzymes (e.g., SOD, GPx, and Trx-1) [27,28], inducing mitophagy to remove damaged mitochondria and reduce ROS production, and altering its activity through direct oxidative modifications (e.g., H_2_O_2_-mediated sulfenylation or glutathionylation) [29,30]. CYP1A1 and oxidative stress form a bidirectional regulatory loop. During polycyclic aromatic hydrocarbon metabolism, CYP1A1 generates large quantities of ROS (H_2_O_2_/superoxide). Excessive AhR activation or substrate load can overwhelm antioxidant defences, inducing lipid peroxidation and oxidative DNA damage [30]. The 3′-UTR rs4646903 polymorphism increases CYP1A1 activity, elevates MDA levels, decreases GPx activity, and enhances susceptibility to oxidative stress [31]. When ROS accumulate excessively, oxidative inactivation of nuclear factor NFI suppresses CYP1A1 promoter activity, forming a negative feedback mechanism that prevents uncontrolled ROS amplification [32]. Under oxidative stress, factors such as ROS, heavy metals, and inflammatory factors strongly induce HMOX-1 expression via the Nrf2-ARE, AP-1, and NF-κB pathways [33]. HMOX-1 exerts protection by catabolizing heme into bilirubin, CO, and free iron—actions that suppress ROS generation, stabilize mitochondrial function, and promote ferritin-mediated iron sequestration. Truncated HMOX-1 isoforms translocate to mitochondria to activate the PGC-1α–NRF1–TFAM axis, promoting mitochondrial biogenesis and mitophagy, or enter the nucleus to enhance AP-1 activity, forming a protective feedback loop against lipid peroxidation and ferroptosis [34]. However, when ferritin buffering is inadequate, excess HO-1–derived free iron catalyzes Fenton reactions, amplifying ROS and turning the pathway into a double-edged sword capable of promoting oxidative stress or apoptosis, as observed in cancer [33]. Oxidative stress and mTOR also form a bidirectional regulatory loop. ROS rapidly inhibit mTORC1 by activating AMPK, oxidizing TSC2, or interfering with Rag–Ragulator lysosomal translocation; ROS can also oxidize Sin1, destabilizing mTORC2 [35]. This suppression relieves ULK1 phosphorylation inhibition, triggering autophagy that clears damaged mitochondria and reduces ROS. Conversely, persistently hyperactive mTOR—due to nutrient surplus or oncogenic signals—increases ROS through enhanced glycolysis and mitochondrial biogenesis (via HIF-1α), repression of FoxO3a-mediated transcription of Mn-SOD and catalase, and elevated NADPH consumption for fatty-acid synthesis. The resulting “ROS↑–mTOR↑” loop inhibits autophagy, amplifies oxidative damage, activates inflammasomes, and drives disease progression in conditions such as COPD, NAFLD, diabetic vasculopathy, and cancer [36]. Inducible nitric oxide synthase acts as a high-output, double-edged mediator during oxidative stress. Once activated by inflammatory cytokines or hyperglycaemia via NF-κB/MAPK signalling, it produces sustained micromolar levels of NO. Initially, NO suppresses the NLRP3 inflammasome and scavenges superoxide, exerting antioxidant effects [37]. However, when NO reacts with concurrently generated O_2_^−^, it forms peroxynitrite (ONOO^−^), which depletes SOD and catalase, induces lipid peroxidation, DNA fragmentation, mitochondrial dysfunction, and ferroptosis. This establishes a self-reinforcing “NO–ONOO^−^–ROS” positive-feedback loop that amplifies oxidative damage and drives apoptosis and tissue inflammation [38]. SOD is the rate-limiting barrier against oxidative stress. Its Cu/Zn- or Mn-containing active sites rapidly convert superoxide into H_2_O_2_, thereby preventing ROS propagation and ONOO^−^ formation [39]. Under oxidative challenge, the ROS–ATM/Cds1 pathway drives nuclear translocation of SOD1, enabling it to act as a transcriptional co-activator that upregulates its own expression and other antioxidant genes, forming a reinforcing protective loop. Population studies show an inverse association between plasma SOD activity and all-cause mortality in older women. At the same time, experimental models demonstrate that decreased SOD correlates with elevated MDA in epilepsy and cancer, marking SOD as a sensitive indicator of oxidative-stress severity [40].

In tissues 4, 9, 13 and 14, the transcript levels of oxidative-stress-related genes rose markedly and these same tissues displayed evident pathological changes, indicating that when the mammary gland is under negative energy balance intracellular ATP falls and AMP accumulates. Consequently, AMPK is transcriptionally up-regulated to launch an energy-saving programme: fatty-acid synthesis is suppressed, fatty-acid oxidation is enhanced and autophagy is activated to recycle intracellular resources. HMOX-1 and SOD are archetypal antioxidant defense genes. During clinical mastitis or LPS challenge, a burst of ROS triggers the Nrf2 pathway; the ensuing rise in HMOX-1 transcription accelerates heme breakdown, generating CO, bilirubin and free iron. Elevated SOD directly scavenges superoxide anions, providing mammary epithelial cells with a first enzymatic line of defense against oxidative insult. CYP1A1, though traditionally viewed as a metabolic enzyme, is also induced by LPS or aflatoxin in bovine mastitis models; its up-regulation suppresses NF-κB and lowers TNF-α/IL-6, thereby exerting both detoxifying and local anti-inflammatory effects. An increase in mTOR mRNA is usually interpreted as heightened anabolic demand. Under negative energy balance or inflammatory conditions, however, AMPK is activated first and transiently restrains mTOR activity via TSC2 phosphorylation. The accompanying rise in mTOR transcript therefore represents a compensatory boost aimed at sustaining protein translation and milk-protein synthesis.

This study has several limitations. Additionally, all specimens were collected from cull cows manifesting severe clinical mastitis at the abattoir; their mammary tissues already exhibited end-stage lesions such as extensive necrosis, calcification, or fibrosis, and the predominant pathogens were acute opportunistic bacteria. Consequently, the observed high-ROS, high-antioxidant-gene profile does not reflect the oxidative-stress status characteristic of the far more prevalent subclinical or mild-to-moderate mastitis encountered in productive dairy herds. Therefore, extrapolating our findings to routine on-farm control scenarios may be biased, and future studies should prospectively collect milk and paired tissue samples from mild cases in both pasture-based and intensive systems to validate the applicability of the proposed biomarkers under real production conditions. The 16S rDNA analysis identified bacteria only to the species level; virulence-factor profiling, resistome analysis, and metagenomics were not performed, preventing assessment of within-species variation in pathogenicity. Future work will integrate phosphoproteomics and metagenomics to dissect host–microbe interactions at a systems level and establish mammary-organoid models using CRISPR-based deletion of nodes such as AMPK and HMOX-1 to define antioxidant therapeutic windows in vitro.

## 5. Conclusions

This study integrated 16S rDNA identification, H&E histopathology, immunohistochemistry, and RT-qPCR/Western blot analyses to systematically characterize the “bacteria–pathology–molecule” triad in bovine mastitis. The pathogen profile was dominated by *Escherichia coli*, *Aeromonas* spp., and *Pseudomonas* spp. Polymicrobial infections contributed to a sequential progression from acute necrotic lesions (haemorrhage, karyorrhexis) to chronic fibro-calcific lesions. Histopathological stages closely corresponded to pathogen virulence profiles, establishing morphological benchmarks for rapid on-farm assessment of infection stage. Quantitative expression profiles of oxidative-stress signalling markers (AMPK/mTOR, CYP1A1, HMOX-1, NOS, and SOD) provide early diagnostic biomarkers and actionable intervention targets. Collectively, this work proposes a three-dimensional model of mastitis—“pathogen-driven, pathologically staged, and molecularly stratified”—offering mechanistic insight and practical tools for precise diagnosis, region-specific management, and targeted antioxidant therapy in dairy cows.

## Figures and Tables

**Table 1:** Primer sequences for real-time polymerase chain reaction.

Gene Name	Primer Sequence (5′–3′)	TM (°C)
*AMPK*	F: GTGGTGACCCTCAAGACCAG	58.3
	R: TTCCGGATGAGGTTTCAGG	
*CYP1A1*	F: TGCAGGAGAACATCCCTACC	56.7
	R: GGTAGGGTGATGAGGTCCAC	
*HMOX-1*	F: CTGACAGCATGCCCCAGGAT	55.1
	R: CTTCTCCTGGGCTCTCTCCT	
*NOS*	F: CCCCAGACAGCTTCTACCT	55.5
	R: TCCTTTGTTACTGCTTCACC	
*mTOR*	F: TGCGGTCACTCGTCGTCAG	60.6
	R: TGCCAGCCTGCCACTCTTG	
*SOD*	F: ATCCACTTCGAGGCAAAGGG	57.8
	R: GTGAGGACCTGCACTGGTAC	
*β-actin*	F: TCTGGCACCACACCTTCTACAAC	60.1
	R: GGACAGCACAGCCTGGAT	

**Table 2:** Bacterial strains isolated from each sample.

Sample ID	The Isolated Bacterial Strain
1	*Escherichia coli*
2	*Escherichia coli*
3	*Pseudomonas, Acinetobacter johnsonii*
4	*Escherichia coli, Pseudomonas, and Aeromonas*
5	*Escherichia coli, Aeromonas*
6	*Pseudomonas peli, Comamonas*
7	*Escherichia coli, Aeromonas salmonicida*
8	*Escherichia coli, Pseudomonas*
9	*Bacillus licheniformis*
10	*Aeromonas*
11	*Pseudomonas guangdongensis, Staphylococcus haemolyticus, and Mammaliicoccus sciuri*
12	No bacterial strain was isolated.
13	*Escherichia coli, Klebsiella pneumoniae*

**Table 3:** Parameters related to the number of immunohistochemically positive cells.

Names of Images	Positive Cells, %	Positive Cell Density, Number/mm^2^	Mean Density	H-Score	IRS
AMPK-1	80.55%	3067	0.1952	178.60	8
AMPK-2	55.17%	1307	0.1497	119.70	6
AMPK-3	83.54%	4977	0.2367	197.31	8
AMPK-4	79.27%	2995	0.2021	224.25	12
AMPK-5	83.43%	4215	0.2288	201.31	8
AMPK-6	73.92%	2591	0.2165	177.22	6
AMPK-7	79.90%	3252	0.1968	210.62	12
AMPK-8	23.88%	1334	0.1371	38.29	2
AMPK-9	84.33%	2555	0.2247	235.32	12
AMPK-10	79.80%	2040	0.2078	195.57	8
AMPK-11	80.35%	2463	0.1995	174.91	8
AMPK-12	87.46%	2859	0.2050	225.00	12
AMPK-13	43.83%	1971	0.1665	78.18	4
AMPK-14	-	-	-	-	-
CYP1A1-1	95.86%	3076	0.1469	226.46	8
CYP1A1-2	93.07%	2044	0.1288	204.69	8
CYP1A1-3	91.94%	4859	0.2234	213.57	8
CYP1A1-4	94.07%	3084	0.1997	262.39	12
CYP1A1-5	96.97%	2443	0.1618	233.43	8
CYP1A1-6	96.03%	2739	0.1786	255.50	12
CYP1A1-7	87.65%	1873	0.1522	178.19	8
CYP1A1-8	95.59%	3311	0.2042	213.55	8
CYP1A1-9	94.28%	2516	0.1560	209.63	8
CYP1A1-10	95.47%	3589	0.1780	248.64	12
CYP1A1-11	96.06%	2671	0.1883	247.85	12
CYP1A1-12	93.45%	2902	0.2290	236.74	12
CYP1A1-13	92.12%	2829	0.2066	207.36	8
CYP1A1-14	-	-	-	-	-
HMOX-1-1	37.18%	1913	0.2546	87.93	4
HMOX-1-2	23.34%	698	0.1912	54.75	2
HMOX-1-3	47.50%	3302	0.2406	107.09	4
HMOX-1-4	35.80%	1807	0.1771	73.54	4
HMOX-1-5	32.57%	2557	0.2378	74.41	4
HMOX-1-6	31.38%	1538	0.2520	71.25	4
HMOX-1-7	74.04%	3437	0.2038	169.70	6
HMOX-1-8	11.94%	707	0.2539	28.47	2
HMOX-1-9	47.43%	2266	0.2407	107.54	4
HMOX-1-10	56.96%	1965	0.2394	131.58	6
HMOX-1-11	39.07%	1663	0.2617	93.90	4
HMOX-1-12	51.78%	2510	0.2535	123.50	6
HMOX-1-13	10.04%	474	0.3138	25.85	3
HMOX-1-14	-	-	-	-	-
mTOR-1	59.81%	2994	0.1493	118.68	6
mTOR-2	19.27%	523	0.1825	42.66	2
mTOR-3	42.43%	2559	0.1746	81.90	4
mTOR-4	75.82%	3046	0.1223	154.01	8
mTOR-5	39.57%	2476	0.1914	79.76	4
mTOR-6	61.65%	3367	0.1548	126.38	6
mTOR-7	41.79%	2814	0.1572	80.34	4
mTOR-8	44.49%	2286	0.1441	78.97	4
mTOR-9	61.45%	2926	0.1657	123.15	6
mTOR-10	63.16%	3225	0.1515	129.79	6
mTOR-11	42.93%	1751	0.1598	82.92	4
mTOR-12	74.41%	3611	0.1560	152.67	6
mTOR-13	48.77%	2005	0.1557	89.94	4
mTOR-14	-	-	-	-	-
NOS-1	30.39%	885	0.1978	54.60	4
NOS-2	20.77%	407	0.1568	35.29	2
NOS-3	35.03%	1288	0.1886	64.11	4
NOS-4	54.77%	976	0.1460	92.97	6
NOS-5	29.48%	909	0.1774	53.04	4
NOS-6	7.50%	187	0.1948	12.23	2
NOS-7	33.89%	487	0.1642	55.47	4
NOS-8	22.56%	1253	0.1441	35.43	2
NOS-9	33.21%	766	0.1953	60.18	4
NOS-10	43.85%	1174	0.2068	86.80	4
NOS-11	36.71%	987	0.1610	64.05	4
NOS-12	35.29%	936	0.1437	61.00	4
NOS-13	0.60%	30	0.2632	1.14	0
NOS-14	-	-	-	-	-
SOD-1	87.33%	2021	0.2091	220.91	12
SOD-2	95.33%	1523	0.2048	267.15	12
SOD-3	75.06%	2308	0.2074	151.74	8
SOD-4	81.21%	2404	0.1452	180.02	8
SOD-5	69.61%	1618	0.2034	145.39	6
SOD-6	58.45%	1211	0.1862	115.29	6
SOD-7	78.31%	2366	0.1694	171.88	8
SOD-8	22.08%	1214	0.1801	40.35	2
SOD-9	86.98%	1244	0.1951	192.10	8
SOD-10	71.89%	1572	0.2084	163.67	6
SOD-11	73.85%	1884	0.1845	153.02	6
SOD-12	90.66%	1256	0.1836	201.02	8
SOD-13	24.80%	1137	0.1878	48.66	2
SOD-14	-	-	-	-	-

## Data Availability

All data and materials are included within the article.

## Supplementary Materials

The following supporting information can be downloaded at: https://www.mdpi.com/article/10.3390/biology15020115/s1, File S1: Original images for Western blot of Figure 5.

## Abbreviations

The following abbreviations are used in this manuscript:

ROS	Reactive oxygen species
NOX	NADPH oxidases
NAC	N-carbamylglutamate
EDTA	Ethylene Diamine Tetraacetic Acid
DAB	Diaminobenzidine
AMPK	Adenosine 5′-monophosphate (AMP)-activated protein kinase
CYP1A1	Cytochrome P450 1A1
HMOX-1	Heme oxygenase 1
SOD	Superoxide dismutase
NOS	Nitric oxide synthase
mTOR	Mammalian target of rapamycin
FBS	Fetal bovine serum
BSA	Bovine serum albumin
HRP	Horseradish peroxidase
