# Supplementary figures and images for: Oxidative-Stress-Mediated AMPK/mTOR Signaling in Bovine Mastitis: An Integrative Analysis Combining 16S rDNA Sequencing and Molecular Pathology
Source: Biology (Basel). 2026 Jan 6;15(2):115. doi: 10.3390/biology15020115 (PMC12837542; doi:10.3390/biology15020115)

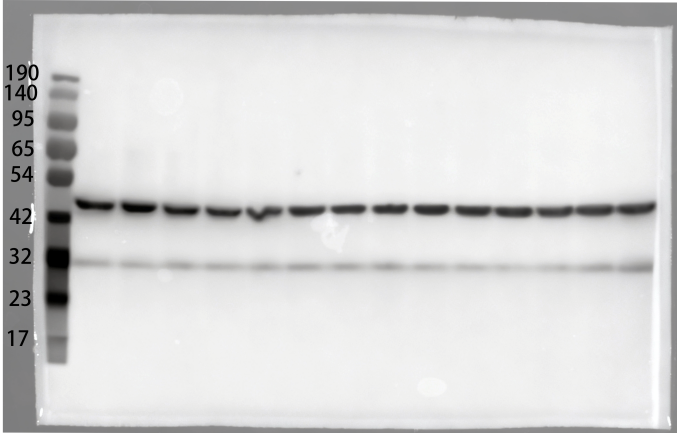

Supplement: Supplementary file 1 [file biology-15-00115-s001.zip › File S1. Original images for Western blot of Figure 5/ACTIN.png]

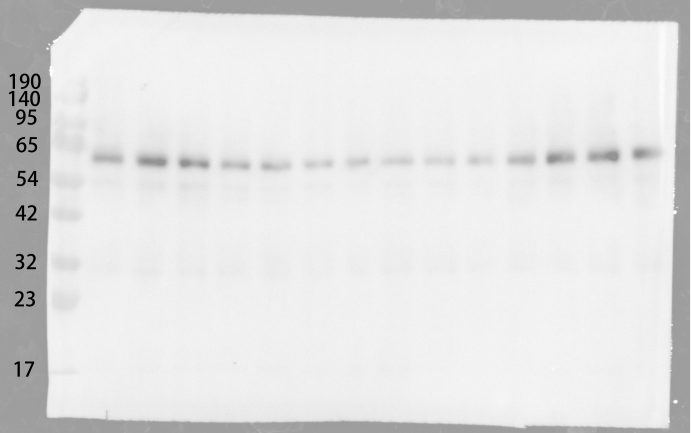

Supplement: Supplementary file 1 [file biology-15-00115-s001.zip › File S1. Original images for Western blot of Figure 5/AMPK.png]

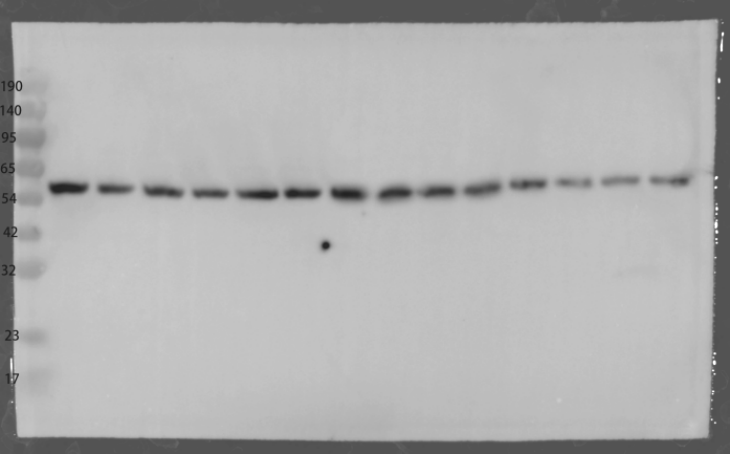

Supplement: Supplementary file 1 [file biology-15-00115-s001.zip › File S1. Original images for Western blot of Figure 5/CYP1A1.png]

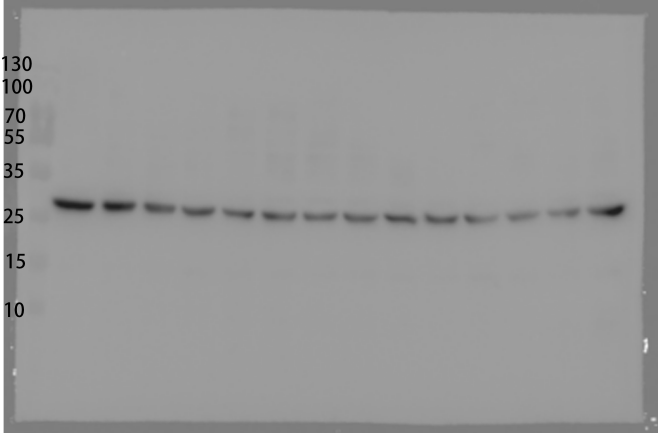

Supplement: Supplementary file 1 [file biology-15-00115-s001.zip › File S1. Original images for Western blot of Figure 5/HMOX-1.png]

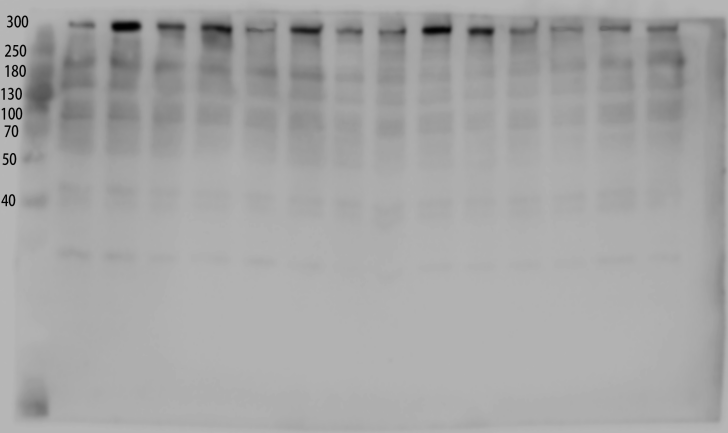

Supplement: Supplementary file 1 [file biology-15-00115-s001.zip › File S1. Original images for Western blot of Figure 5/MTOR.png]

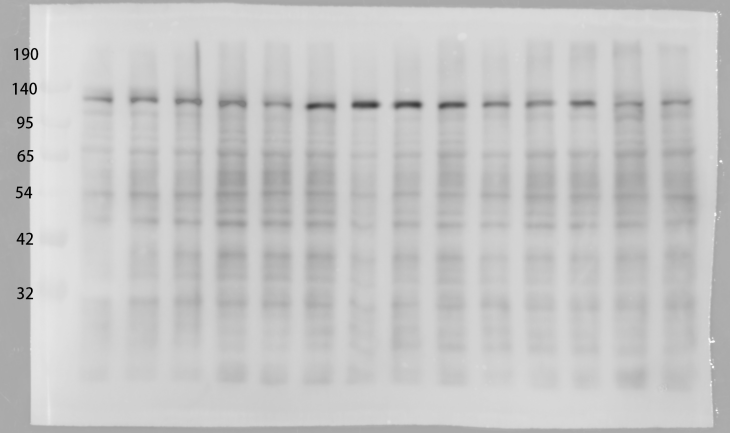

Supplement: Supplementary file 1 [file biology-15-00115-s001.zip › File S1. Original images for Western blot of Figure 5/NOS.png]

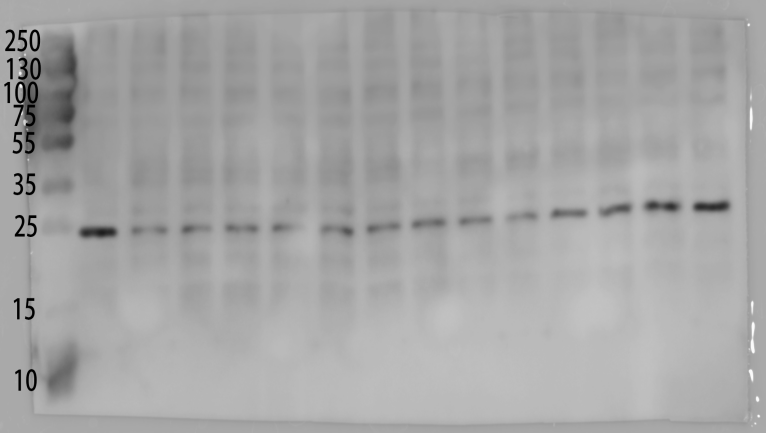

Supplement: Supplementary file 1 [file biology-15-00115-s001.zip › File S1. Original images for Western blot of Figure 5/SOD.png]
